# Selection and identification of high‐affinity aptamer of Kunitz trypsin inhibitor and their application in rapid and specific detection

**DOI:** 10.1002/fsn3.2729

**Published:** 2022-01-19

**Authors:** Yunxiang Bao, Dengzhao Zhu, Yang Zhao, Xinzhu Li, Chunmei Gu, Hansong Yu

**Affiliations:** ^1^ College of Food Science and Engineering Jilin Agricultural University Changchun China; ^2^ Division of Soybean Processing Soybean Research & Development Center Chinese Agricultural Research System Changchun China

**Keywords:** aptamer, biosensor, colorimetric detection, gold nanoparticles, graphene oxide‐SELEX, KTI

## Abstract

Kunitz trypsin inhibitor (KTI), a harmful protein, seriously affects food hygiene and safety. Therefore, a sensitive, efficient, and rapid method for KTI detection is urgently needed. Aptamers are short and single‐stranded (ss) DNA that recognize target molecules with high affinity. This work used graphene oxide‐SELEX (GO‐SELEX) to screen KTI aptamers. The positive and reverse screening was designed to ensure the high specificity and affinity of the selected aptamers. After 10 rounds of screening, multiple nucleic acid chains were obtained, and the chains were sequenced. Three aptamers with better affinity were obtained, and the values of the dissociation constant (*K*_d_) were calculated to be 52.6 nM, 22.7 nM, and 67.9 nM, respectively. Finally, a colorimetric aptamer biosensor based on gold nanoparticles (AuNPs) was constructed. The biosensor exhibited a broader linear range of 30–750 ng/ml, with a lower detection limit of 18 ng/ml, and the spiked recovery rate was between 98.2% and 103.3%. This experiment preliminary demonstrated the potential of the application of KTI aptamer in the real sample tests.

## Graphical abstract

Nonfixed aptamer selection method: GO‐SELEX was used to screen KTI aptamers. Aptamers with *K*_d_ values of 52.45 nmol/L (AKti‐5), 22.56 nmol/L (AKti‐10), and 67.94 nmol/L (AKti‐15) were obtained. Based on the selected KTI aptamers, a colorimetric aptamer biosensor based on AuNPs was established, with a detection limit of 17.8508 ng/ml. The linear range was 30–750 ng/ml.

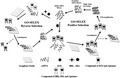

## 1 INTRODUCTION

Kunitz trypsin inhibitor (KTI), one of the main antinutritional factors of soybean (Maetens et al., 2018), has a severely hazardous influence on the digestive system and pancreas tissues (Liener, 1995). Its content in soybeans is 1.4% (Bode & Huber, 1992). Therefore, establishing comprehensive, specific, and efficient detection methods of KTI in soybeans appears to be particularly necessary (Alves et al., 2010). Enzyme‐linked immunosorbent assay (ELISA), as a common method to detect KTI, has significant specificity and efficiency (Chen, Huang, et al., 2014; Chen, Wang, et al., 2014; Hei et al., 2012). However, the signal molecules (antibody) of ELISA are not easy to obtain. Xu et al. (2016) developed a strategy to detect KTI by preparing KTI monoclonal antibodies. However, due to the complex composition of the food matrix, it is hard to distinguish substances with similar structures. ELISA often has false‐positive or false‐negative results (Farzam et al., 2017; Mani et al., 2016). Therefore, the development of detection methods with cost and time advantages is particularly important.

Aptamers are functional single‐stranded oligonucleotides that fold into a unique three‐dimensional conformation based on secondary structures, which can recognition target through geometric matching or multiple noncovalent interactions (Bunka & Stockley, 2006). Compared with antibodies, aptamers have many advantages: high affinity, strong specificity, wide target range, low cost, small molecular weight, easy synthesis and modification, low immunogenicity, low toxicity, and good stability (Keefe et al., 2010). Therefore, aptamers are expected to function as an alternative to protein‐based antibodies. Aptamers are widely applied in many fields such as chemical analysis, protein function research, biomedical research, and clinical research (Khati, 2010; Tang et al., 2012; Yu et al., 2015). Nguyen and Jang (2020) established an aptamer biosensor based on label‐free liquid crystal (LC) to detect arsenic (III) ions (As^3+^) in aqueous solutions, which had a detection limit of 50 nM. Cheng Yang et al. (2011) constructed a colorimetric aptamer biosensor based on unmodified gold nanoparticles (AuNPs) to detect ochratoxin A (OTA), whose detection limit was 20 ng/ml. Thus, aptamers as recognition molecules are promising for the development of biosensors.

The vitro screening methods for aptamers include magnetic‐bead SELEX, solid‐phase carrier‐SELEX and capillary electrophoresis‐SELEX. In recent years, methods for screening aptamers using graphene oxide (GO) have gradually emerged. GO has a strong adsorption effect on single‐stranded deoxyribonucleic acid (ssDNA) (Báez et al., 2021). Therefore, it can be used to screen aptamers (GO‐SELEX). Gao et al. (2016) obtained the aptamers against gonyautoxins 1 and 4 by magnetic‐bead SELEX and GO‐SELEX, respectively. Though comparing the two methods, they concluded that GO‐SELEX was more advantageous in the aptamers screening for small molecule substances. Nguyen et al. (2014) obtained ten aptamers that can bind to three pesticides by GO‐SELEX. The affinity of these aptamers with pesticides was all within the range of 10–100 nM.

**SCHEME 1:**
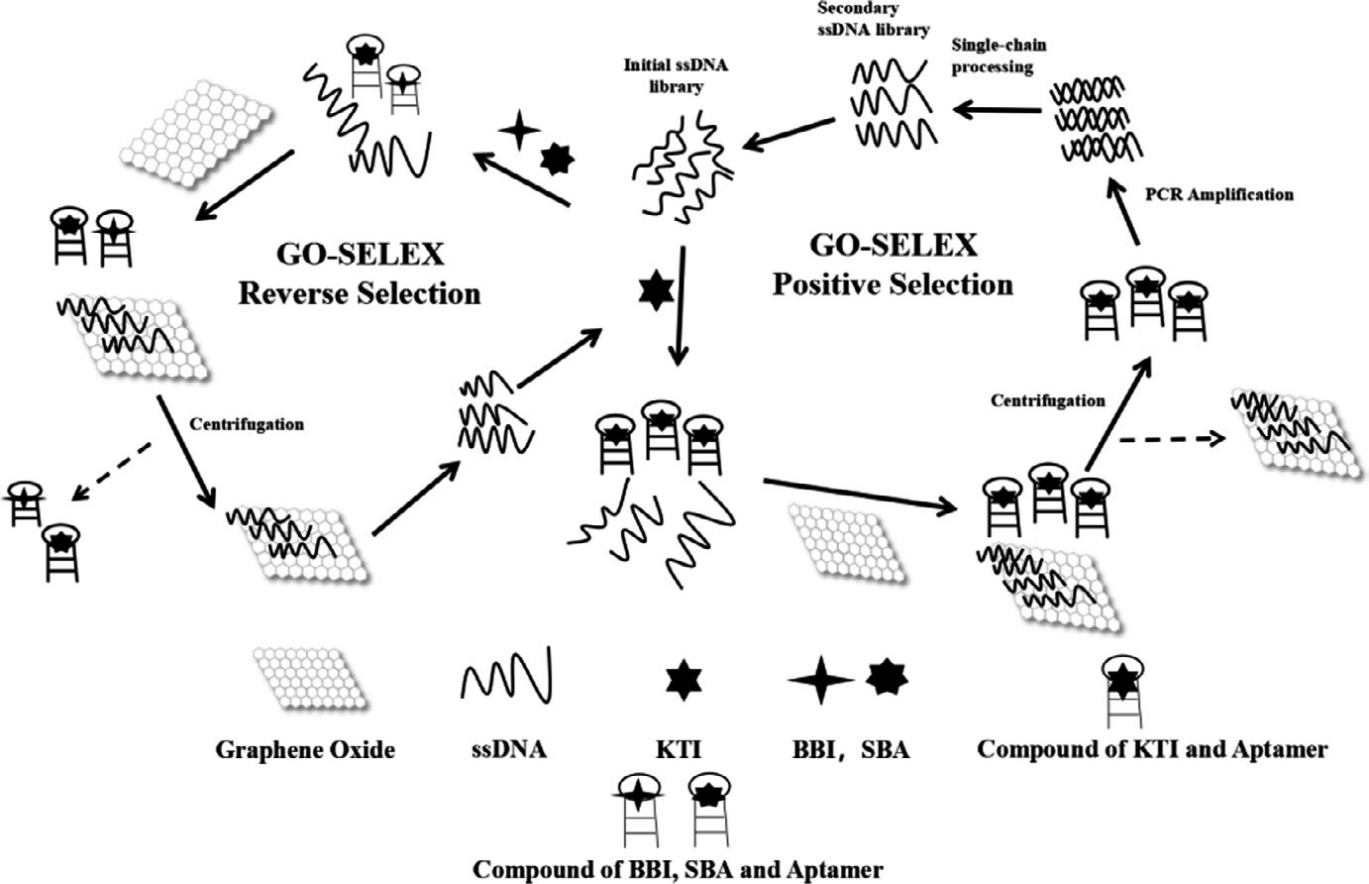
The mechanism and process of selection of KTI aptamers by GO‐SELEX method

To date, aptamers against numerous targets have been selected by systematic evolution of ligands by exponential enrichment (SELEX) and used for designing aptamer biosensors, such as metal ions, small molecules, bacteria, cells, and tissues. (Bock et al., 1992; Ferreira et al., 2008; Ylera et al., 2002). Nevertheless, KTI aptamers have not been studied yet. By referring to Park and Narges Hedayati (Hedayati et al., 2021; Park et al., 2012; Wu et al., 2011), an aptamer screening method was constructed in this study. GO‐SELEX was used to screen KTI aptamers. Scheme 1 shows the entire screening process: Redundant ssDNA was adsorbed and removed by GO. Furthermore, the screening process was monitored by fluorescence assay. As the screening process continues, the sequences that bind to KTI with high affinity are continuously enriched, and KTI aptamers are finally obtained. KTI aptamers were used as the signal molecule to construct a biosensor to detect KTI, which provides a new idea for KTI detection (Ma et al., 2018).

## 2 MATERIALS AND METHODS

### 2.1 Materials

The starting material for the program was a random library of oligonucleotides. The library consists of a primer binding region of 20 nucleotides on both sides and a random region of 40 nucleotides (40N) in the middle. The sequence was 5′‐FAM‐AGCAGCACAGAGGTCAGATG‐N40‐CCTATGCGTGCTACCGTGAA‐3′. Forward primer (FP) was 5′‐AGCAGCACAGAGGTCAGATG‐3′, reverse primer (RP) was 5′‐TTCACGGTAGCACGCATAGG‐3′, and the complementary sequence of forward primer (FP‐c) was 5′‐CATCTGACCTCTGTGCTGCT‐3′. Those primers and random ssDNA library were dissolved in TE buffer (pH = 8.0) (Qi et al., 2012).

KTI, BBI, and SBA standards were purchased from Sigma‐Aldrich (USA), and GO and streptavidin magnetic beads were purchased from Xianfeng Nanomaterials Technology Co., Ltd (Nanjing, China). 6×Loading buffer (DNA) single stain, 2×Taq plus PCR Colorless Mix, and SYBR Green I 10000×DMSO were purchased from Dingguo Changsheng Biotechnology Co., Ltd (Beijing, China). HEPES, Tris, agarose, and DL2000 Marker were purchased from Baori Biotech Co., Ltd (Beijing, China). Glycogen (for nucleic acid precipitation) was purchased from Biyuntian Biotechnology Co., Ltd (Shanghai, China). Gel Extraction Kit (DNA recovery kit) was purchased from Omega Bio‐Tek (USA). All the other reagents were analytically pure and purchased from Sinopharm Chemical Reagent Co., Ltd (Beijing, China).

### 2.2 Instruments and equipment

Aluminum gradient PCR instrument (Vapo, Protect) was purchased from Eppendorf (Germany), and electrophoresis system (JY‐SPC) was purchased from Junyi Dongfang electrophoresis equipment (Beijing, China). Gel imaging system (2500 R) was purchased from Precision Instrument Co., Ltd (Shanghai, China). Ultrahigh‐speed refrigerated centrifuge (3‐18K) was purchased from Sigma Company (Germany). Analytical balance (SOP) was purchased from Sartorius Scientific Instrument Co., Ltd (Beijing, China). UV spectrophotometer (NanoDrop‐2000 trace) was purchased from Thermo Scientific Company (USA). Multifunctional microplate reader (VICTOR Nivo) was purchased from PerkinElmer (USA).

### 2.3 Optimization of the amount of GO

0.2 nmol ssDNA library was treated to make its structure reach the most stable state (Wang, Lyu, et al., 2020; Wang, Wang, et al., 2020): ssDNA was heated at 95°C for 10 min, immediately taken for an ice bath for 10 min, and finally stored at 25°C. GO solutions with different mass ratios (the mass ratios of ssDNA to GO were 1:50, 1:100, 1:150, 1:200, 1:300, 1:400, and 1:500 (w/w)) were mixed with the treated ssDNA library. The solutions were incubated at 37°C, 120 r/min for 2 h, and then centrifuged at 14,000 r/min for 15 min to collect the supernatant. The fluorescence intensity (*F*) of the supernatant was determined by a multifunctional microplate reader. The fluorescence quenching efficiency reflected the combination of GO and ssDNA using the following equation, Equation (1):

(1) $$Q\left(\%\right)=\left(f_{0}‐f\right)/f\times 100$$

where *Q* is the fluorescence quenching efficiency, *f*_0_ is the fluorescence intensity of the control group without GO, and *f* is the experimental group. The adsorption saturation curve with GO concentration as the abscissa and fluorescence quenching efficiency as the ordinate was plotted.

### 2.4 Screening process of KTI aptamers

Firstly, the random ssDNA library, FP‐C, and biotin‐labeled RP were treated according to the method in 2.3 to maximize the stability of the structure.

#### 2.4.1 Positive screening

About 10 nmol KTI was mixed with ssDNA library and incubated at 25°C on a shaker for 60 min to make KTI fully bind to ssDNA. Then, GO solution was added to the solution and incubated at 25°C on a shaker for 60 min to adsorb the ssDNA unbound to KTI. The solutions were then centrifuged at 14,000 r/min for 15 min to remove the precipitation. The remaining GO in the supernatant was eliminated by a 3 M sodium acetate solution (pH = 5.2). The solution was left standing at −20°C for 30 min to remove residual GO. After adding ice ethanol and nucleic acid coprecipitation agent to the solutions, the solution was then centrifuged at 12,000 r/min for 15 min to collect ssDNA. The ssDNA was washed with 75% ice ethanol and then dried by vacuum freeze‐drying. Finally, the ssDNA precipitation was redissolved in 20 μl double distilled water (dd water).

#### 2.4.2 Amplification of ssDNA

ssDNA screened out in each round was amplified by PCR. PCR system includes the following: template DNA 5 μl (10 μmol/L), FAM‐labeled FP 2 μl (20 μmol/L), biotin‐labeled RP 2 μl (20 μmol/L), PCR Mix (including DNA polymerase) 25 μl, and 16 μl ddH_2_O. The conditions are as follows: predenaturation at 95°C for 10 min, denaturation at 95°C for 30 s, annealing at 60.3°C for 30 s, extension at 72°C for 30 s, the number of cycles set as the optimal number of cycles, extension at 72°C for 7 min, and preservation at 4°C. The amplification products were verified by 3% agarose gel electrophoresis. Amplification products in the gel were recovered and purified by using the gel extraction kit.

#### 2.4.3 Preparation of secondary library

One hundred microliter streptavidin magnetic beads were taken and rinsed by 200 μl 0.01 mol/L PBS three times. Then, DNA amplified by PCR was added and incubated at 37°C on a shaker for 30 min. The precipitation was collected by centrifuging at 14,000 r/min for 15 min. Then, the streptavidin magnetic beads were rinsed with 0.01 mol/L PBS three times. Single‐chain treatment was carried out by adding 200 μl 200 mmol/L NaOH. The mixed solution was centrifuged at 14,000 r/min for 15 min to collect the supernatant, and the ssDNA in the supernatant was recovered by ice ethanol and nucleic acid coprecipitation agent.

#### 2.4.4 Reverse screening

To improve the specificity of KTI aptamer, BBI (Bowman–Birk inhibitor) and SBA (Soybean agglutinin) were used as the reverse screening targets. The ssDNA library was mixed with BBI and SBA and incubated at 25°C on a shaker for 60 min. GO solution was added to adsorb ssDNA that was not bound to the reverse screening targets. The precipitation was retained and redispersed into the solution.

### 2.5 Monitoring of the screening process

The fluorescence intensity of ssDNA in each round was measured by a multifunctional microplate reader, and the recovery rate was calculated using the following equation, Equation (2):

(2) $$R\left(\%\right)=\left(f_{a}/f_{b}\right)\times 100$$

where *R* is the recovery rate of ssDNA binding to KTI, *f_a_* is the fluorescence intensity of ssDNA before the screening, and *f_b_* is the fluorescence intensity of ssDNA after the screening. As the screening progressed, ssDNA that binds to KTI was constantly enriched until the recovery rate kept stable.

### 2.6 High‐throughput sequencing and sequence analysis

The amplified products of PCR were sequenced on Illumina HiSeq 2000 sequencer after the final round of screening. The sequences with an occurrence number greater than 10 were selected for further analysis. The homology analysis was performed by MEGA7 software on the multiple aptamer chains obtained by sequencing to divide the sequences into some families (Hedayati et al., 2021). Online analysis software RNA Structure 6.0 was then used to calculate the minimum Gibbs‐free energy variable (ΔG) and predict the secondary structure of the sequences in each family. The representative sequence of each family was selected as the candidate aptamers and synthesized by Shenggong Bioengineering Co., Ltd (Shanghai).

### 2.7 Investigation of affinity and specificity between KTI and candidate aptamer

The affinity of aptamers to target is usually represented by the dissociation constant (*K*_d_), which is determined by fluorescence analysis in this work (Eissa & Zourob, 2017). GO was added to the solutions of 5′FAM‐labeled aptamer with different concentrations (0, 20, 100, 200, 400, 600 nM) and incubated in the dark for 60 min. Then, 1 μmol/L KTI solutions were added and incubated for 2 h at room temperature in the dark. After centrifuging at 13,000 r/min for 10 min, the supernatant was collected, and the fluorescence value was determined by a multifunctional microplate reader. The nonlinear binding curve and *K*_d_ values of the aptamer to target were fitted by GraphPad Prism 7 software.

The specificity of Akti‐10, Akti‐5, and Akti‐15 was investigated by fluorescence analysis (Hu et al., 2018). BBI and SBA can also be used to testify the specificity of aptamers obtained in this study. Firstly, KTI, BBI, and SBA standard solutions were added to incubate with candidate aptamers, respectively. Then, the free redundant ssDNA was removed by GO, and the fluorescence value was determined. The experiments were repeated three times.

### 2.8 Application of KTI aptamers

Using KTI aptamer as the signal molecule, a colorimetric aptamer biosensor based on AuNPs was constructed to detect KTI. The general process of detection is as follows: Firstly, 10 µl KTI aptamer solution (8 μmol/L) was added to 100 µl AuNP solution (2.5 μmol/L) and incubated at 37°C for 3 h. Then, the solutions were mixed with KTI standard of different concentrations and incubated for 1 h. Finally, 10 µl NaCl solution (750 mmol/L) was added to the solution. The color change in the solutions was recorded, and the absorbance value of solutions at 520 nm and 656 nm was determined. The KTI concentration was represented by *A*_656 nm_/*A*_520 nm_, and the standard curve of KTI concentration was established.

The feasibility of the KTI aptamer biosensor in real simple was analyzed by soybean milk. Firstly, 1% soybean milk buffer solution was prepared with ddH_2_O. Then, the buffer solution was used to dilute KTI to 100, 200, and 500 nmol/L. Finally, the aptamer biosensor constructed in this study was used to detect KTI content in buffer solution. The spiked recovery rate was determined by comparing the measured value with the actual concentration. Three parallel tests were performed for each concentration, and the relative standard deviation (RSD) was calculated to illustrate the precision of the detection method. RSD was calculated using the following equation, Equation (3):

(3) $$\text{RSD}\;\left(\%\right)=100\times \sqrt{\frac{\sum_{i=1}^{n}{\left(x_{i}‐\bar{x}\right)}^{2}}{n‐1}}$$

where RSD is the relative standard deviation, *x* is the measured value, $\bar{x}$ is the average, and *n* is the number of measurements.

## 3 RESULTS AND DISCUSSION

### 3.1 Optimization of the amount of GO

**FIGURE 1:**
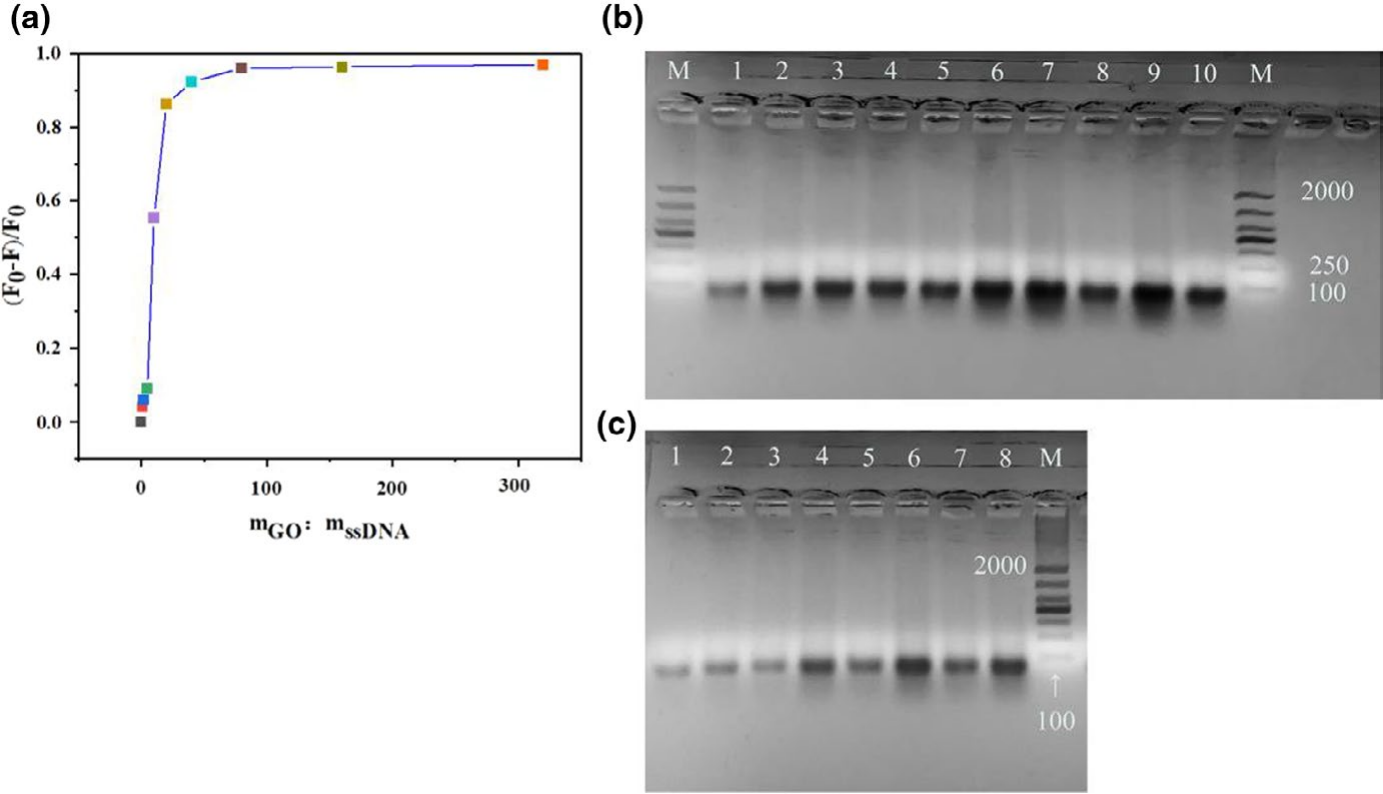
Optimization of screening conditions. Optimization of the adsorption ratio of the library to GO (a). Optimization of annealing temperature (b). The band M was used to represent DL2000 DNA Marker; Bands 1–10 were used to represent 55.2, 55.7, 56.4, 57.2, 58.2, 59.2, 60.3, 61.2, 62.0, and 62.5°C, respectively. Optimization of the number of PCR cycles (c). The band M was used to represent DL2000 DNA Marker; Bands 1–8 were used to represent 4, 6, 8, 10, 12, 14, 16, and 18 cycles of PCR, respectively

GO has the ability to adsorb ssDNA, but this ability has little effect on double‐stranded DNA (dsDNA). Thus, GO was used to absorb and remove ssDNA with weak binding ability to KTI in the ssDNA library (Xing et al., 2019). Excessive GO will affect the combination of the aptamers to KTI, while insufficient GO will lead to low screening efficiency. Therefore, the amount of GO was optimized to ensure the quality and efficiency of aptamer screening. As shown in Figure 1a, the fluorescence quenching efficiency had a growing trend with the increase in GO. When the mass ratio of GO to the library was 150:1 (w/w), the fluorescence quenching efficiency reached the highest and kept saturated, indicating that GO had completely absorbed ssDNA. Therefore, the mass ratio of GO to the library was set as 150:1 (w/w).

### 3.2 In vitro selection of KTI aptamers

In each round of screening, a large amount of ssDNA with a weaker affinity to KTI would be lost, resulting in the remaining ssDNA in the library being insufficient to support the next round of screening. Therefore, PCR was taken to amplify ssDNA in each round. Amplifying under optimal conditions can avoid nonspecific amplification, so optimizing the annealing temperature and the number of PCR cycles is necessary. Figure 1b showed the annealing temperature optimization: Band 7 was the brightest and unique band, located at 80 bp, indicating that Band 7 was the most successfully amplified band. Therefore, the annealing temperature was set as 60.3°C. Figure 1c showed the optimization of the number of PCR cycles: Band 6 was the brightest band, located at 80 bp, indicating that Band 6 was the most successfully amplified band. Therefore, the optimal number of cycles for amplification was 14.

**FIGURE 2:**
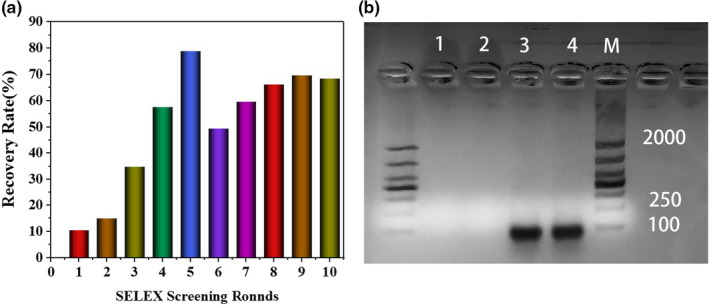
The recovery rate of ssDNA binding with KTI after each round of screening (a). The recovery rate was determined by recording the fluorescence value of ssDNA before and after screening. Electrophoretogram of PCR product in the final round of screening (b). Band M represented DL2000 DNA Marker; Bands 1 and 2 were negative control, and Bands 3 and 4 were the screening product

The ssDNA recovery rate of each round was determined to monitor the enrichment degree of the ssDNA with a strong binding ability to KTI. The ssDNA recovery rate of each round is shown in Figure 2a. The recovery rate showed a gradual rising trend initially, which illustrated that the ssDNA with high affinity to KTI was constantly enriched. From the 6th round, the trend showed a decline fluctuation but soon resumed upward. This phenomenon is because the reverse screening removed a lot of redundant ssDNA in the library. The trend of recovery rate kept stable in the 10th round, indicating that the enrichment degree of KTI aptamer reached saturation. So the screening was terminated in the 10th round. KTI aptamer was verified by agarose gel electrophoresis to ensure their quantity and quality. The result is shown in Figure 2b. The screening product band was bright, located at 80 bp, and no band appeared in Bands 1 and 2, indicating that the quality was qualified.

### 3.3 Sequencing results and analysis

**FIGURE 3:**
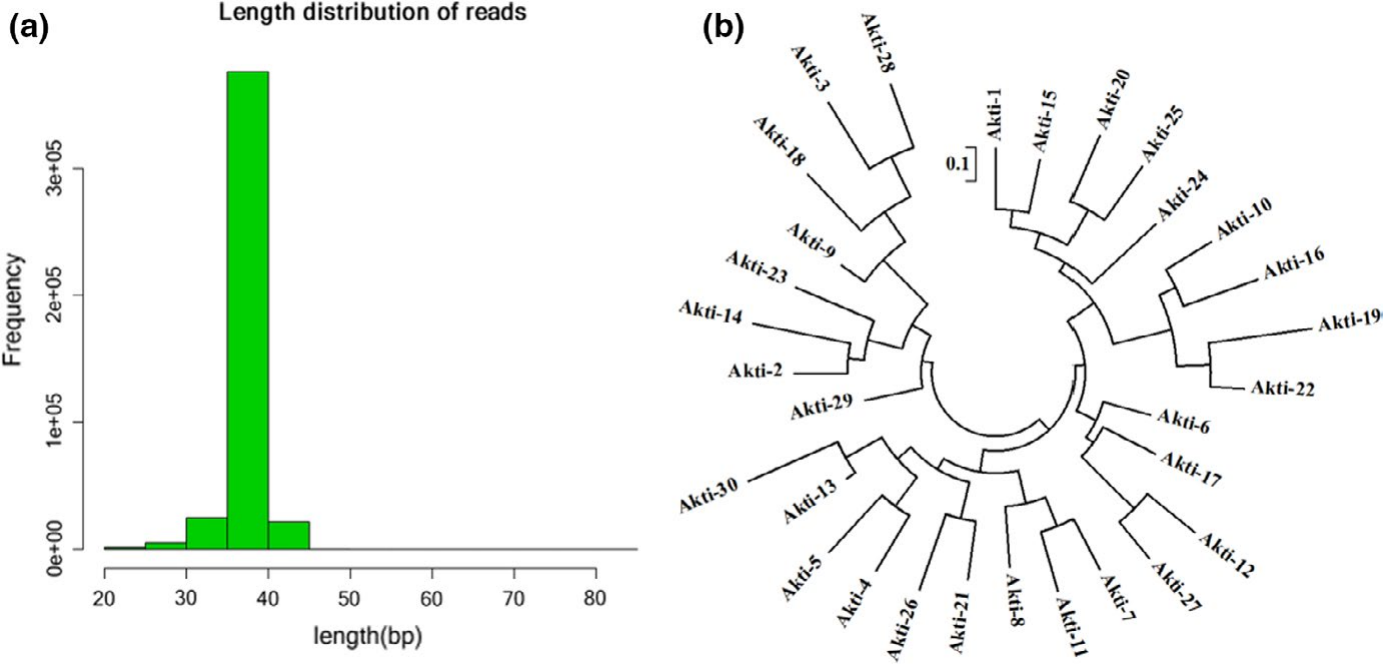
Distribution of the length of the aptamer sequences after high‐throughput sequencing (a). Sequences with s length of 40 nt occupied the main part. Phylogenetic tree of the candidate sequences (b). The similarity of these 30 sequences was 68.92%

The enriched KTI aptamers were sequenced. As shown in Figure 3a, sequences with s length of 40 nt occupied the main part, and sequences with high occurrence frequency accounted for about 6.7%. Sequences whose occurrence frequency was greater than 10 were selected and named from Akti‐1 to Akti‐30. The phylogenetic tree of 30 sequences was constructed by MEGA6, and the result is shown in Figure 3b. The similarity of 30 sequences was 68.92%, indicating a high similarity. According to the phylogenetic tree, 30 sequences were divided into 6 families. Then, RNA Structure 6.0 was used to simulate the minimum Gibbs‐free energy (Δ*G*) of the sequences in each family. The smaller the Δ*G*, the more stable the secondary structure was, and it is more conducive to the combination of aptamer and target (Chen, Huang, et al., 2014; Chen, Wang, et al., 2014). Following the principle of high occurrence frequency and small Δ*G*, the representative sequences were finally selected in each family, which were Akti‐15, Akti‐9, Akti‐11, Akti‐5, Akti‐28, and Akti‐10, as shown in Table 1.

**TABLE 1:** Representative sequence and its Gibbs‐free energy in different families

Family	Sequence	ΔG
A	Akti‐15	−9.8121
B	Akti‐9	−10.7121
C	Akti‐11	−14.4721
D	Akti‐5	−12.6621
E	Akti‐12	−14.5821
F	Akti‐10	−12.8521

### 3.4 Investigation of binding affinity and specificity

**FIGURE 4:**
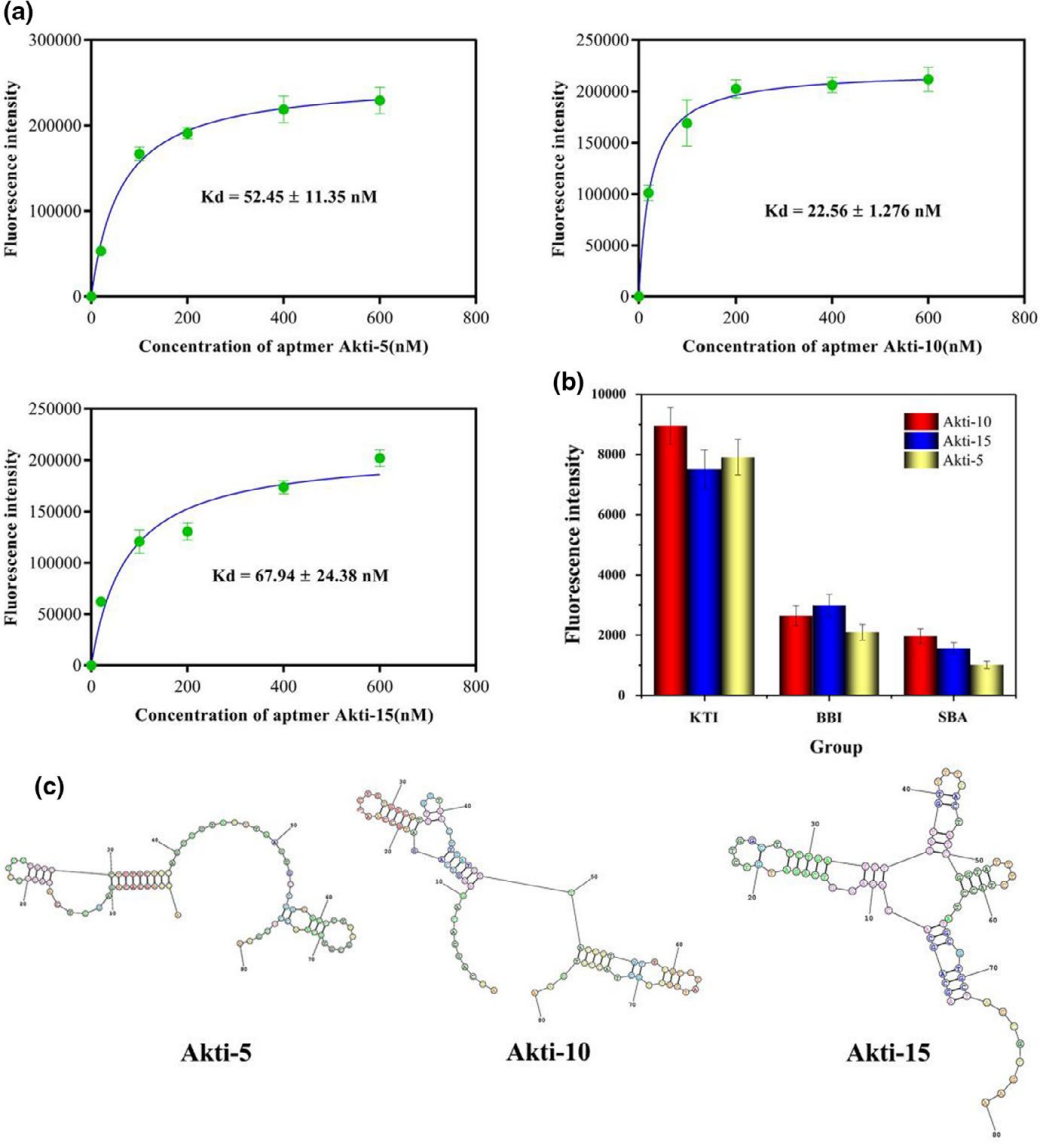
The saturation curves with *K*_d_ values of aptamer Akti‐5, Akti‐5, and Akti‐10 (a). The binding ability of ssDNA was determined, and the binding saturation curve was plotted by recording the changes in fluorescence values before and after the binding of ssDNA with GO. Specificity of Akti‐5, Akti‐15, and Akti‐10 binding to KTI (b). The specificity of KTI was tested by comparing the binding ability of BBI and SBA to the aptamer. The predicted secondary structures of aptamer Akti‐5, Akti‐5, and Akti‐10 (c)

A fluorescence assay was used to evaluate the binding affinity of candidate aptamers. GraphPad Prism 7 software was used to fit the binding curve and the *K*_d_ value between each sequence and KTI. The smaller the *K*_d_ value was, the higher the affinity was. As shown in Table 2, the *K*_d_ value of each candidate sequence was within the measurement range, indicating that these sequences had a high affinity with KTI. The *K*_d_ values of Akti‐5, Akti‐15, and Akti‐10 were lower than other candidate sequences, which were 52.6 ± 11.4 nM, 67.9 ± 24.4, and 22.7 ± 1.3 nM, respectively. The binding curves of Akti‐5, Akti‐15, and Akti‐10 are shown in Figure 4a.

**TABLE 2:** Dissociation constant of KTI aptamers

KTI aptamer	Sequence (5′‐3′)	*K*_d_ (nmol/L)
Akti‐5	CTTCGTATCCTGTGCTGCAGTTGTTTTTAAGTGCTGGGAG	52.45 ± 11.35
Akti‐9	CTGGTGTTTTCTTTACGCATAACAAGGCCCCTGAGAGGCT	133.8 ± 74.18
Akti‐10	CCAACCTGTGGCATTTGGTAACTTGATCTTACGGTGTTCG	22.56 ± 1.276
Akti‐11	GGTCGTGAGGCCCACCGCTGAGCTCTAGTATCGCTCGGCG	116.3 ± 45.84
Akti‐15	TCTGGCTTCTGATCTGATGTTTTTACTATCGGTATTTCTA	67.94 ± 24.38
Akti‐28	TGTACCGCCCACCTGCCGCTAACGGCGGTGCTGCGGTGCG	164.4 ± 89.84

Due to the lack of research on KTI aptamer, the aptamers' *K*_d_ values in this research can only be compared with other materials' aptamers. For example, Hu et al. (2018) obtained aptamers specific to acrylamide (AA) with the *K*_d_ values of 115.2 nM and 17.2 nM. Wang, Lyu, et al. (2020) and Wang, Wang, et al. (2020) obtained aptamers specific to diethylstilbestrol (DES) with the *K*_d_ values of 126.3 nM and 98.5 nM. Their results are slightly higher than KTI aptamers in this work. It indicates that the aptamers obtained in this work have a high affinity with KTI.

The structure of BBI is much similar to KTI. SBA accounts for 10% of soy protein. Therefore, in traditional detection methods of KTI, eliminating the interference of SBA and BBI has been a difficult problem to solve. BBI and SBA were used to testify the specificity of KTI aptamers obtained in this study. The results are shown in Figure 4b, Akti‐5, Akti‐15, and Akti‐10 all had high specificity with KTI. The secondary structures of Akti‐5, Akti‐15, and Akti‐10 were shown in Figure 4c. In conclusion, Akti‐10 has the lowest *K*_d_ value and the highest specificity, which indicated that Akti‐10 could be chosen for application in the real sample tests.

### 3.5 Colorimetric detection of KTI with screened aptamers

#### 3.5.1 Mechanism of KTI aptamer biosensor based on AuNPs

**SCHEME 2:**
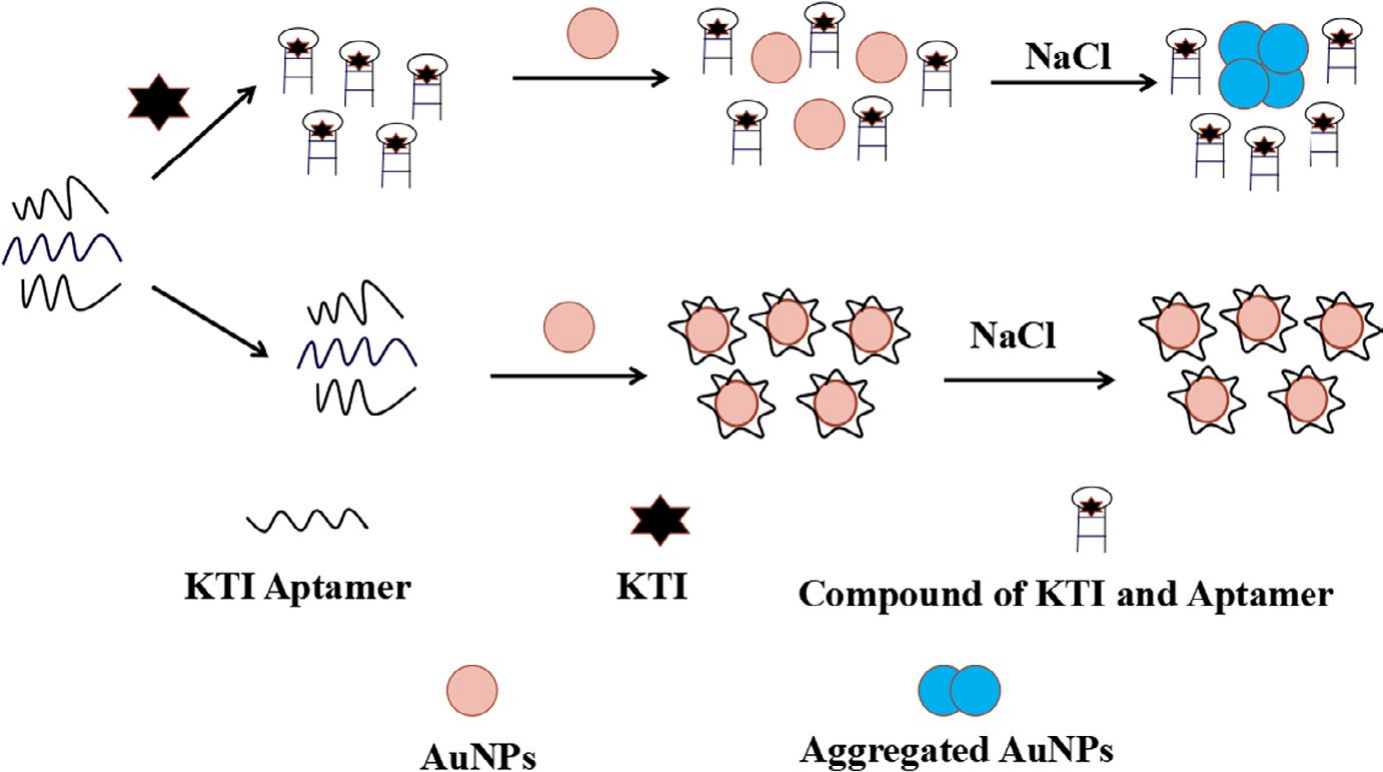
The mechanism of detecting KTI by colorimetric aptamer biosensor based on AuNPs

In order to verify the practical application of the aptamers, a colorimetric aptamer biosensor based on AuNPs was constructed. The general process was shown in Scheme 2. Due to electrostatic interaction, AuNPs in the solution will aggregate after adding NaCl, and the color of the solution will change from red to blue (Erman et al., 2021). ssDNA can combine with AuNPs to protect AuNPs from aggregation. Consequently, AuNP solution will keep stable and red color after the NaCl intervention. However, when KTI is added to the solution, it will form a unique three‐dimensional structure with the aptamer. Consequently, the AuNPs will lose the protective effect of the aptamer to resist NaCl induction, and the color will change from red to blue (Yang et al., 2020).

#### 3.5.2 Characterization of AuNPs

**FIGURE 5:**
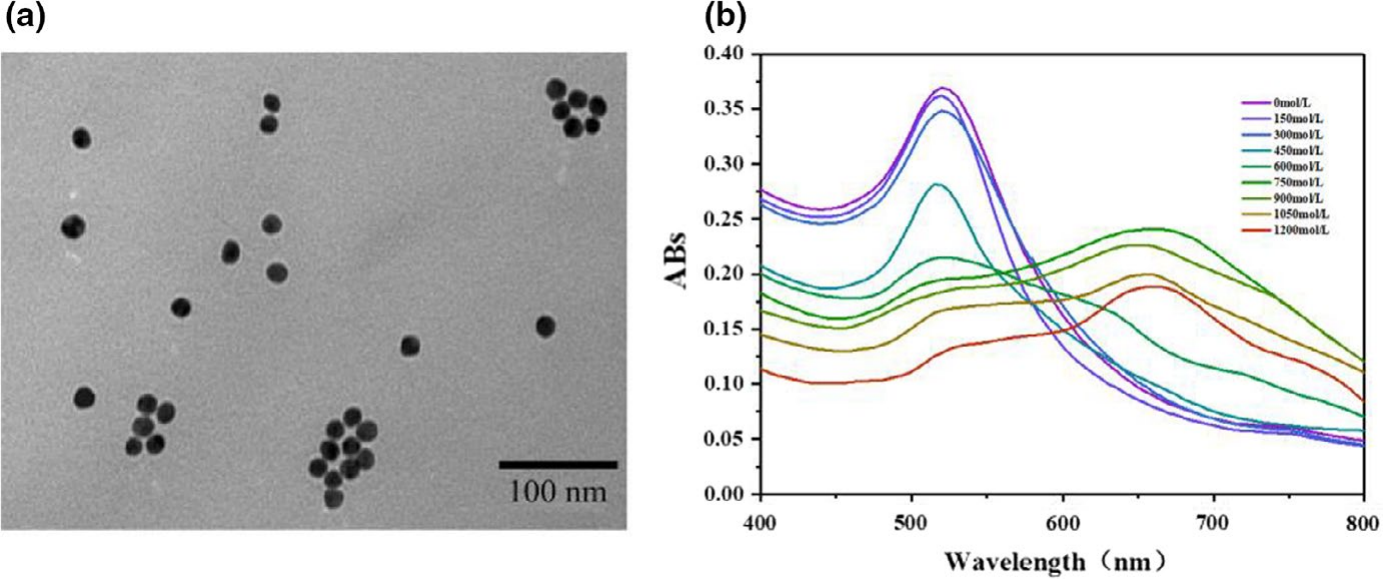
The construction of a colorimetric biosensor based on AuNPs. TEM image of AuNPs (a). The color variation and ultraviolet absorption spectrum of the AuNP solution intervened by NaCl (b). The relationship between the degree of AuNP aggregation and solution absorbance was studied by analyzing the changes of its ultraviolet absorption spectrum

The size and morphology of AuNPs were characterized by transmission electron microscopy (TEM) (Wang, Lyu, et al., 2020; Wang, Wang, et al., 2020). As shown in Figure 5a, the diameter of AuNPs ranged from 12 to 16 nm, and the particles were dispersed and uniform in size.

#### 3.5.3 The degree of AuNP aggregation

The UV absorption spectrum of AuNP solution inducted by different concentrations of NaCl was detected. The result was shown in Figure 5b. As the concentration of NaCl increases, the AuNP solution's color gradually turned to grayish‐blue from wine‐red, and the absorbance value at 520nm decreased constantly. Furthermore, a new characteristic peak appeared at 656 nm, and the peak value increased continuously. This phenomenon was caused by the Plasmon resonance changes from the aggregation of AuNPs (Mugisawa & Sawada, 2008). Therefore, the degree of AuNP aggregation was represented by *A*_656nm_/*A*_520nm_.

#### 3.5.4 Optimization of the assay conditions

**FIGURE 6:**
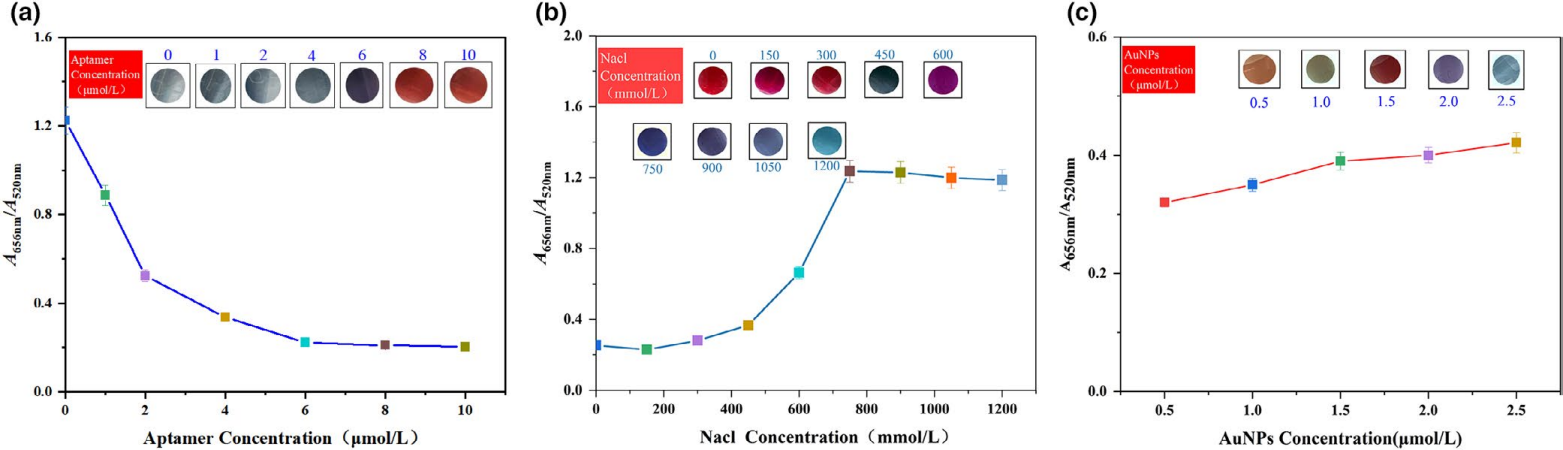
Optimization of KTI aptamer biosensor. The assay conditions were optimized by colorimetric method. The aggregation degree of AuNPs was indicated by *A*_656nm_/*A*_520nm_. Optimization of KTI aptamer concentration (a); optimization of NaCl concentration (b); optimization of AuNP concentration (c)

The assay conditions, including the concentration of aptamer, NaCl, and AuNPs, affect the stability and precision of the KTI aptamer biosensor. Therefore, the concentrations of aptamer, NaCl, and AuNPs were optimized firstly. The optimization results were shown in Figure 6a–c. With the increase in aptamer concentration, the degree of AuNP aggregation showed a gradually decreasing trend and finally kept steady from 8 μmol/L. With the increase in NaCl concentration, the degree of AuNP aggregation showed a gradually increasing trend and finally kept steady from 750 mmol/L. With the increase in AuNP concentration, the degree of AuNP aggregation showed a gradually increasing trend and finally reached its maximum in 2.5 μmol/L. Based on the above results, the concentration of NaCl, AuNPs, and aptamer was eventually optimized to be 750 mmol/L, 2.5 μmol/L, and 8 μmol/L.

#### 3.5.5 Construction of KTI standard curve

**FIGURE 7:**
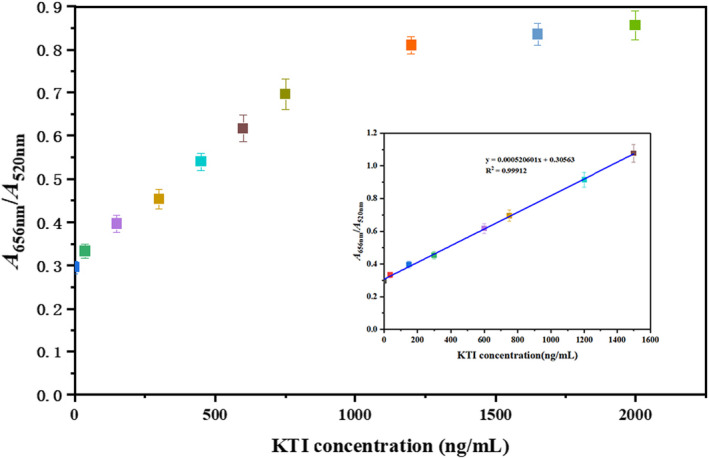
The linear range and KTI standard curve of KTI aptamer biosensor. The linear range and KTI standard curve were optimized by colorimetric method. The aggregation degree of AuNPs was indicated by *A*_656nm_/*A*_520nm_

The linear relationship between KTI concentration and the degree of AuNP aggregation was investigated. The result is shown in Figure 7. KTI concentration had an excellent linear relationship with the degree of AuNP aggregation from 30 to 750 ng/ml, and the linear correlation coefficient (*R* ^2^) was 0.999. The limit of detection (LOD) was calculated to be 18 ng/ml, which was an ultramicro level.

#### 3.5.6 Practical feasibility of KTI aptamer biosensor

Soybean milk was used to test the feasibility of the KTI aptamer biosensor in a real sample test. The results are shown in Table 3. The spiked recovery rate of KTI in soybean milk samples was between 98.2% and 103.3%, which is in line with the relevant requirements of food physical and chemical testing in China's national standard GB/T 27404‐2008, indicating that this method can be applied in the real sample test. The RSD of the KTI aptamer biosensor was between 2.39% and 4.38%, indicating that the precision of this detection method was good.

**TABLE 3:** Standard recovery and RSD of KTI aptamer biosensor

Number	Concentration (ng/ml)	Average recovery rate (%)	RSD (%)
1	50	103.3	2.39
2	100	98.2	2.51
3	500	101.3	3.39
4	1000	102.9	4.38
5	1500	99.3	3.07

In addition, the aptamer biosensor in this work also has many merits. (1) Fast detection speed: The time in an overall reaction is short. (2) Low cost: The synthetic cost of AuNPs and aptamers is extremely low. (3) Low requirements for experimental conditions: The requirement of expensive instruments and skilled technicians is unnecessary. (4) Flexibility: It can be detected qualitatively by a color change and quantitatively by detecting absorbance.

## 4 CONCLUSIONS

In summary, to improve the detection specificity, we successfully selected KTI aptamers by GO‐SELEX in this work. GO was used to absorb and remove the redundant ssDNA in the selecting process, and the enrichment degree was monitored by fluorescence detection. Through 10 rounds of selecting, KTI aptamers bound to KTI were successfully obtained, and their *K*_d_ values were 52.6 ± 11.4 nM (Akti‐5), 22.7 ± 1.3 nM (Akti‐10), and 67.9 ± 24.4 nM (Akti‐15). Using KTI aptamer as the detection probe, a colorimetric aptamer biosensor based on AuNPs was established. The linear range of the biosensor was 30~750ng/ml, and the detection limit was 18 ng/ml. Soybean milk was used to test the feasibility of the KTI aptamer biosensor in the real sample. The spiked recovery rate of KTI was between 98.2% and 103.3%. Furthermore, this colorimetric aptamer biosensor showed higher specificity than the methods proposed before by our group.

## CONFLICT OF INTEREST

The authors declare that they have no competing interests.

## AUTHOR CONTRIBUTIONS

**Yunxiang Bao:** Conceptualization (lead); Data curation (lead); Formal analysis (lead); Investigation (lead); Methodology (lead). **Dengzhao Zhu:** Funding acquisition (equal); Investigation (equal); Methodology (equal). **Yang Zhao:** Formal analysis (equal); Investigation (equal); Project administration (equal). **Xinzhu Li:** Conceptualization (equal); Data curation (equal). **Hansong Yu:** Funding acquisition (equal); Resources (equal); Supervision (equal).

## Data Availability

Research data are not shared.
